# *Enterococcus* and cancer: from mechanistic insights to clinical translation

**DOI:** 10.3389/fimmu.2026.1882184

**Published:** 2026-08-19

**Authors:** Haojuan Feng, Xiujuan Huang, Yongju Zhuang, Lianfang Pu, Ruofan Dong, Ping Ren

**Affiliations:** 1 Wuxi School of Medicine, Jiangnan University, Wuxi, Jiangsu, China; 2 Department of Obstetrics, Affiliated Hospital of Jiangnan University, Wuxi, Jiangsu, China

**Keywords:** anti-tumor activity, enterococcus, immune microenvironment, immunotherapy, microbiome-based therapeutics

## Abstract

Despite advances in traditional therapies, the global burden of malignant tumors remains substantial. Although immunotherapy has achieved remarkable breakthroughs by activating the host immune system, its clinical benefits are limited to a subset of patientsowing to low response rates and drug resistance. Emerging evidence highlights the pivotal role of the gut microbiota in determining immunotherapy outcomes. Within this ecosystem, the genus *Enterococcus* acts as a “double-edged sword”: whereas certain strains can enhance antitumor immunity, other species notably *Enterococcus faecalis*, drive tumor progression by inducing DNA damage and inflammatory cascades. Therefore, elucidating the intricate interactions between *Enterococcus* and the host immune system is imperative for optimizing immunotherapeutic strategies and developing novel microbiome-based therapies.

## 1 Introduction

Tumor immunotherapy, exemplified by PD-1/PD-L1 inhibitors, overcomes immune suppression to activate T cells against cancer cells (1). However, clinical efficacy varies considerably among patients. Emerging research has identified the gut microbiota, often regarded as the body’s largest immune-regulating organ, as a critical determinant of immunotherapy response.

The trillions of microbes residing in the gut continuously regulate systemic immune homeostasis through metabolites (e.g., short-chain fatty acids) and immune signaling (2). Among these, certain *Enterococcus* strains are key immunomodulatory bacteria that can modulate the immune microenvironment through multiple pathways. For example, they can migrate to the tumor microenvironment, activate dendritic cells and CD8+ T cells, and enhance immunotherapy efficacy (3). Additionally, these strains activate innate antitumor immunity by modulating the PD-L2/RGMb pathway and secreting bacterial membrane vesicles.

Herein, this review discusses the mechanisms by which *Enterococcus* and its metabolites modulate the immune system, with the goal of identifying potential pathways beneficial for tumor immunotherapy. Additionally, we comprehensively review existing research regarding the impact of *Enterococcus* on malignant tumors. Through an in-depth analysis of strain-specific characteristics, we seek to elucidate the dual roles of *Enterococcus* in both promoting and inhibiting tumorigenesis and progression. Furthermore, we discuss strategies to mitigate the risks associated with *Enterococcus* pathogenicity and antibiotic resistance, thereby laying a solid foundation for its safe and effective clinical translation.

## 2 The impact of enterococcus on human immune system

**Figure 1:**
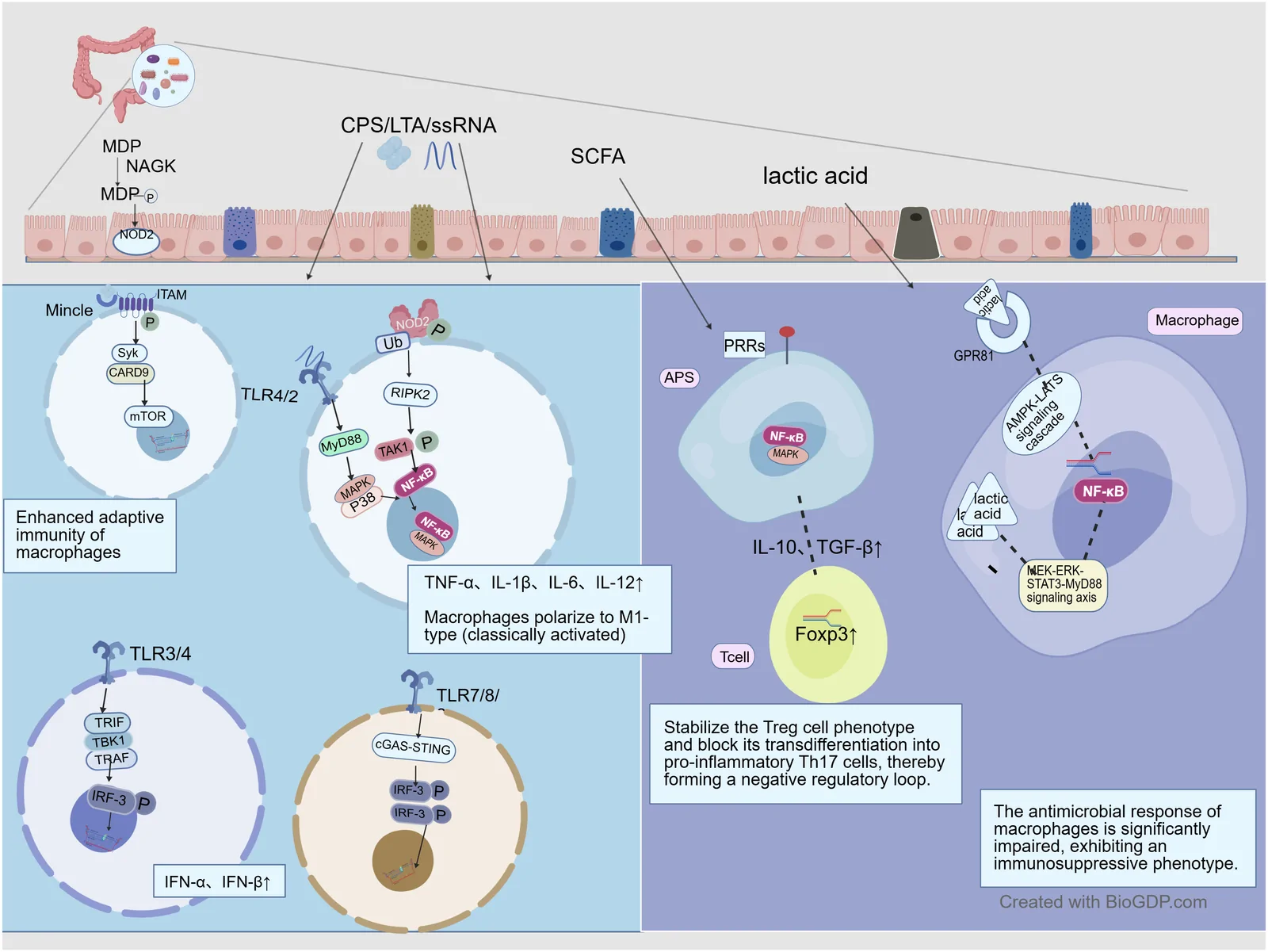
Schematic representation of the mechanisms by which Enterococcus-derived components and metabolites regulate the host immune microenvironment (42). The illustration depicts the dual role of gut microbiota patterns (e.g., MDP, CPS/LTA/ssRNA) and metabolites (lactic acid) in modulating macrophage polarization and T cell differentiation via distinct signaling cascades. **(A)** Pro-inflammatory and Immune-Activating Pathways (Left panel): Bacterial components are recognized by receptors such as TLR4/2, TLR3/4, TLR7/8, and NOD2, triggering downstream signaling cascades involving NF-κB, MAPK, and IRF-3. This leads to the release of pro-inflammatory cytokines (TNF-α, IL-1β, IL-6), promoting M1 macrophage polarization and enhancing adaptive immunity. **(B)** Anti-inflammatory and Immunosuppressive Pathways (Right panel): Lactic acid binds to GPR81 to inhibit the AMPK-LATS axis, thereby suppressing the MEK-ERK-STAT3-MyD88 pathway and impairing the antimicrobial response of macrophages. Concurrently, the secretion of IL-10 and TGF-β, stabilizing the Foxp3+ regulatory T cell (Treg) phenotype and forming a negative regulatory loop. Legend: blue backgrounds indicate pro-inflammatory effects. Purple backgrounds indicate immunosuppressive effects. Solid arrows represent interactions supported by direct experimental evidence; dashed arrows represent hypothetical models derived from current literature. (Created with BioGDP.com) (41).

*Enterococcus* comprises Gram-positive cocci that are widely distributed in nature and the intestines of animals. Although traditionally considered it a harmless commensal flora, an increasing body of evidence has confirmed that *Enterococcus* is actually a “double-edged sword”—it can regulate immune responses and maintain host health on one hand, and may also participate in disease pathogenesis by inducing chronic inflammation or mediating immune escape on the other (4, 5). Its impact on the host immune system is characterized by complex, multi-level features that are highly strain-specific and environment-dependent (6, 7). In recent years, advances in genomic sequencing and molecular biology have progressively illuminated the precise molecular mechanisms underlying *Enterococcus*–host immune interactions, offering fresh perspectives on the regulatory dynamics of this commensalism–pathogenesis balance (**Figure 1**).

### 2.1 Activation of pro-inflammatory response and innate immunity

*Enterococcus* (e.g., *Enterococcus faecalis* CECT7121) activates immune cells to induce the IL-12-Th1-IFN-γ axis, thereby enhancing innate immunity and phagocytic function. *Enterococcal* cell wall components (particularly peptidoglycan and lipoteichoic acid [LTA]) are recognized by pattern recognition receptors (PRRs) such as TLR2 and NOD2 on macrophages and dendritic cells, thereby initiating MyD88-dependent signal transduction cascades that activate NF-κB and MAPK pathways and substantially upregulate interleukin-12 (IL-12) production (7–10). IL-12 subsequently directs naïve CD4^+^ T cell differentiation into Th1 cells via T-bet-mediated transcriptional programming. These activated Th1 cells produce abundant interferon-γ (IFN-γ), which reciprocally activates macrophages to enhance their microbicidal capacity and stimulate the secretion of proinflammatory mediators, including tumor necrosis factor-α (TNF-α) and nitric oxide (NO), thereby establishing a self-amplifying positive feedback loop (11). A robust Th1-type immune response generally correlates with favorable prognoses in patients with various cancers (12).

*Enterococcus* (such as *E. faecalis*) induces macrophage pyroptosis and inflammatory responses. Membrane vesicles (MVs) released by *Enterococcus* carry various bioactive molecules (including lipoteichoic acid [LTA], DNA, and muramyl dipeptide [MDP]), which are endocytosed and recognized by intracellular NOD2 recognizes peptidoglycan fragments in MVs, whereas TLR2 recognizes LTA, synergistically activating the NOD-like receptor signaling pathway and the NF-κB pathways (13). NF-κB nuclear translocation initiates the transcription of target genes, including pro-IL-1β and pro-IL-18, and triggers NLRP3 inflammasome assembly.

Subsequent caspase-1 activation cleaves gasdermin D (GSDMD) and pro-cytokines, resulting in pyroptotic cell death and the robust release of mature proinflammatory mediators such as IL-1β, TNF-α, and IL-6 (14–16). This process amplifies the innate immune response through inflammatory cascades but may also cause tissue damage under specific pathological conditions. Macrophage pyroptosis induced by *Enterococcus* membrane vesicles (MVs) exerts a ‘double-edged sword’ effect by reshaping the tumor immune microenvironment. Whereas moderate activation can enhance anti-tumor immunity, excessive activation leads to the sustained release of proinflammatory cytokines, such as IL-1β and TNF-α, creating a chronic inflammatory microenvironment that may promote tumor progression (17).

Translocation of *Enterococcus gallinarum* drives Th17 differentiation and autoantibody production, thereby triggering autoimmune pathology. Certain enterococcal strains, notably *E. gallinarum*, exhibit barrier-crossing capacity and can disseminate to extraintestinal sites such as the liver and spleen. At these locations, they promote local Th17 cell polarization and autoantibody generation (e.g., anti-RNA antibodies), consequently triggering or exacerbating autoimmune responses (3, 18, 19). In the context of tumor immunotherapy, oral administration of MRx0518 enables its surface flagellin to bind to Toll-like receptor 5 (TLR5), thereby activating the NF-κB pathway and promoting dendritic cell maturation. This initiates a T-cell-mediated immune response against tumors. Consequently, it exhibits significant synergistic antitumor efficacy when combined with immune checkpoint inhibitors, such as anti-PD-1/PD-L1 antibodies. Routy et al. reported that *E. gallinarum* and several other *Enterococcus* species were relatively more abundant in patients responding to immune checkpoint inhibitors (ICIs) (20). Oral prophylaxis with the commensal strain *E. gallinarum* MRx0518, isolated from the healthy human gut, yielded robust antitumor efficacy in murine models of breast, lung, and renal cancers (21, 22).

Emerging data indicate that *Enterococcus* species might drive epigenetic rewiring of myeloid progenitors, thereby promoting trained immunity. In murine models of intestinal inflammation, *enterococci* (e.g., *Enterococcus faecalis*) can traverse the compromised intestinal barrier and translocate to the bone marrow. Granulocyte-macrophage progenitors (GMPs) in the bone marrow recognize enterococcal antigens via the surface C-type lectin receptor Mincle (23). This interaction activates the downstream Syk-mTOR signaling cascade, orchestrating epigenetic reprogramming marked by H3K4me3 histone modifications. Upon secondary exposure to pathogenic stimuli, these “trained” macrophages demonstrate a heightened and accelerated inflammatory response, substantially improving host defense against secondary infections (24).

*Enterococcus* induces cross-reactive immunity through molecular mimicry, offering new insights for tumor immunotherapy. Specific antigens expressed on the surface of *Enterococci*, for instance, phage-encoded tail tape measure proteins on *Enterococcus hirae*, share structural similarity with host tumor antigens. Through the molecular mimicry mechanisms, these antigens can elicit cross-reactive immune responses (25). This process may disrupt autoimmune tolerance, causing immune attacks targeting *Enterococcus* to be erroneously directed toward tumor tissue, or conversely, causing antitumor immune responses to target *Enterococcus*-colonization sites. In patients with renal cell carcinoma and lung cancer, the presence of enterococcal prophages in feces and the expression of TMP cross-reactive antigens in tumors are closely associated with long-term efficacy of PD-1 blockade therapy. This provides new insights into the development of *Enterococcus*-based tumor vaccines or immunotherapies.

### 2.2 Immune suppression and anti-inflammatory regulation

Certain *Enterococcus* strains, such as *E.* durans M4-5, induce the production of IL-10/TGF-β to promote regulatory T cell (Treg) differentiation, thereby maintaining intestinal immune homeostasis. Specifically, *E.* durans M4–5 produces butyrate and other short-chain fatty acids (SCFAs), which facilitate Treg differentiation and stimulate the production of anti-inflammatory cytokines (*e.g.*, IL-10). This effectively suppresses excessive inflammatory responses and preserves gut immune homeostasis (26, 27). Antigen-presenting cells (APCs) secrete large amounts of interleukin-10 (IL-10) and transforming growth factor-β (TGF-β) (9). In a TGF-β-dominated microenvironment, naïve T cells begin to express the lineage-specific transcription factor Foxp3, a definitive marker of Treg cell identity that confers immunosuppressive functions. IL-10 further stabilizes the Treg cell phenotype by enhancing TGF-β signaling and blocking transdifferentiation into proinflammatory Th17 cells (28). This establishes a negative regulatory loop that prevents tissue damage caused by excessive immune activation.

*Enterococcus faecalis* inhibits NF-κB via dual lactate signaling pathway, thereby attenuating macrophage antimicrobial responses E. faecalis efficiently produces substantial amounts of lactate via lactate dehydrogenase. The accumulated lactate inhibits NF-κB activation through two distinct signaling routes (7, 29, 30), thereby suppressing the release of inflammatory cytokines, controlling excessive or chronic inflammation and potentially disrupting immune tolerance inducing autoimmunity. The first is the extracellular receptor pathway: extracellular lactate binds to the G-protein-coupled receptor GPR81, triggering the AMPK-LATS signaling cascade, which restricts NF-κB transcriptional activity through phosphorylation-mediated inactivation of Yes-associated protein (YAP). The second is the intracellular metabolic pathway: lactate internalized via MCT-1 accumulates intracellularly, disrupting the integrity of the MEK-ERK-STAT3-MyD88 signaling axis and blocking the cascade signals required for NF-κB nuclear translocation. These two pathways ultimately converge at NF-κB inactivation, resulting in significantly attenuated macrophage antimicrobial responses and an immunosuppressive phenotype. Genetic functional studies have confirmed that NF-κB activation can only be fully restored when both MCT-1 and GPR81 are simultaneously knocked down, indicating functional complementarity and redundancy between the two pathways (31).

### 2.3 Bidirectional immunomodulatory effects of enterococcus

Unlike *E. faecalis* CECT7121, although other E. faecalis strains can also activate the TLR2 receptor, they subsequently upregulate IRAK-M, a key negative regulator of TLR signaling in human periodontal ligament cells. This upregulation suppresses the production of chemokines such as IL-8, thereby blocking excessive neutrophil recruitment and preventing tissue-damaging inflammation (29). Furthermore, surface proteins of specific probiotic strains (e.g., Ornithine transcarbamylase [OTC] of *E. faecium* WEFA23) are recognized by the TLR2 receptor on macrophages (30). However, unlike the pro-inflammatory cascade induced by pathogenic bacteria, these proteins effectively suppress excessive activation of the NF-κB/MAPK pathway (32). This “mild activation” mode possesses dual functionality: it enhances macrophage phagocytic clearance of pathogenic bacteria while avoiding tissue damage caused by “cytokine storms”.

## 3 Mechanisms of action in tumor immunotherapy

Under normal physiological conditions, the immune system recognizes and eliminates malignantly transformed cells through immune surveillance mechanisms. However, tumor cells can construct an immunosuppressive microenvironment through multiple pathways to evade immune attack. These mechanisms include the upregulation of immune checkpoint molecules (*e.g.*, PD-L1), the secretion of immunosuppressive cytokines such as TGF-β and IL-10, and the induction of regulatory T cell (Treg) infiltration (33, 34). The core objective of tumor immunotherapy is to reactivate antitumor immune cell function and systematically reverse tumor immune evasion mechanisms (35).

The core strategies of tumor immunotherapy mainly encompass four categories: immune checkpoint inhibitors block pathways such as PD-1/PD-L1, CTLA-4, and TIM-3 to relieve tumor-mediated T cell suppression and reverse T cell exhaustion (36). Adoptive cell therapy (ACT) strengthens antitumor immune responses by expanding or genetically modifying autologous immune cells ex vitro (e.g., CAR-T, TCR-T, and TIL therapies) followed by reinfusion (37). Cancer vaccines (*e.g.*, dendritic cell vaccines and mRNA vaccines) aim to present tumor-specific antigens, break immune tolerance, and induce durable antigen-specific immunity. Additionally, cytokines and immunomodulators (*e.g.*, IL-2, IFN-α, and Bacillus Calmette-Guérin [BCG]) provide foundational support for antitumor immunity through nonspecific immune system activation (38, 39).

Currently, immunotherapeutic strategies represented by immune checkpoint blockade (ICB) and adoptive cell transfer (ACT) have achieved breakthrough clinical progress, inducing long-term regression or even functional cure in patients with certain refractory tumors (40). These therapeutic approaches reshape antitumor immune responses and restore the immune system’s ability to recognize and eliminate tumor cells through distinct molecular mechanisms, such as relieving T cell exhaustion, enhancing effector cell cytotoxic activity, and optimizing tumor antigen presentation.

## 4 Dual effects of *Enterococcus* on malignant tumor therapy

**Figure 2:**
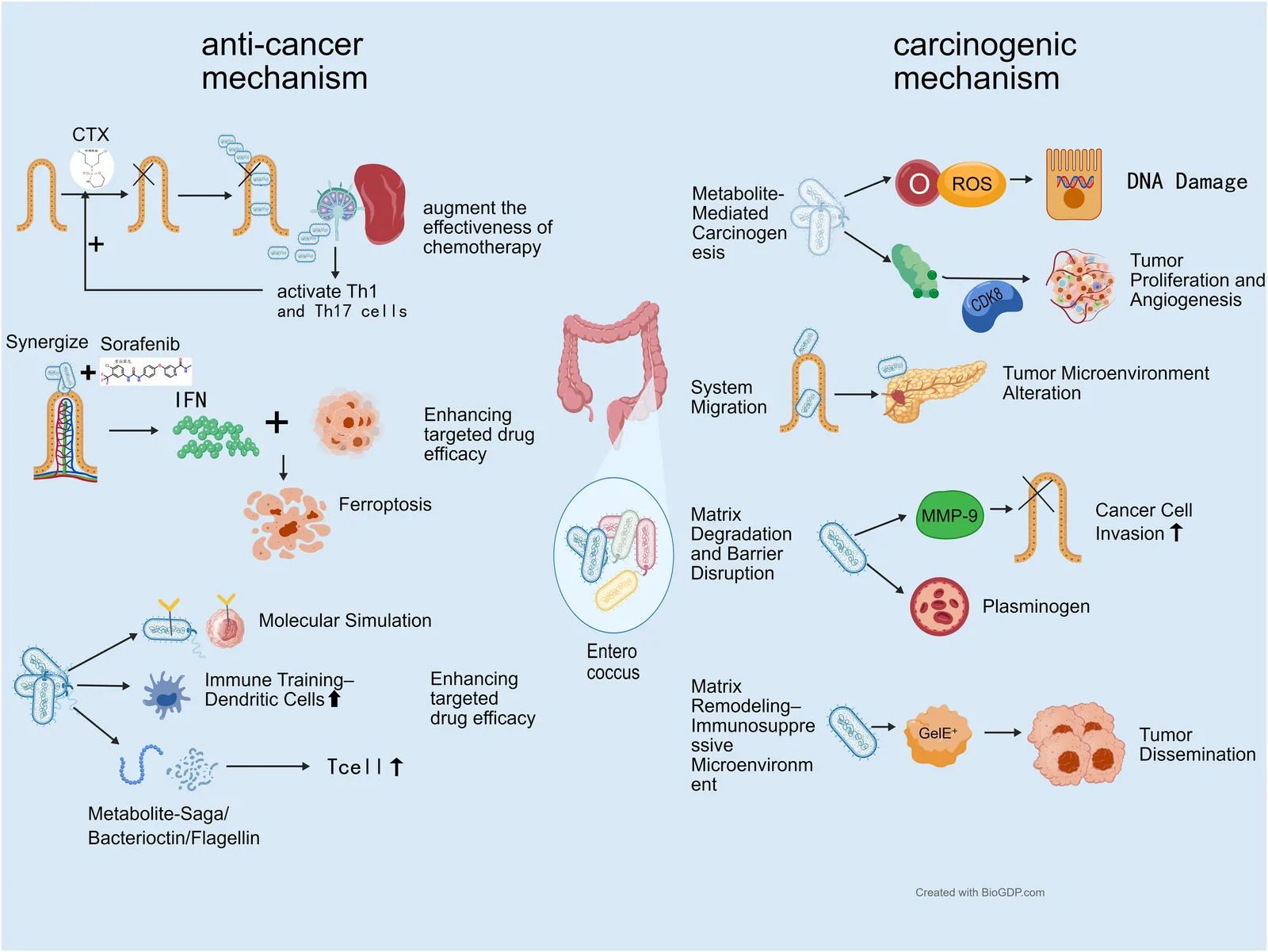
Bidirectional effects of Enterococcus on tumorigenesis (Created with BioGDP.com) (41).

The distinction between the protumorigenic and antitumorigenic effects of *Enterococcus* is not determined by species alone, but rather depends on the interplay among strain genotype, host immune status, and therapeutic intervention. Consequently, *Enterococcus* exhibits a highly strain-specific “double-edged sword” effect in malignant tumor therapy (**Figure 2**). On one hand, specific commensal or probiotic strains (such as *E. hirae*, *E. lactis* MNC-168, and *E. faecium*) can mildly activate immune receptors such as TLR2 via specific surface proteins, or modulate the microenvironment through translocation, enzyme production, lactic acid secretion (30, 31, 42, 43). These mechanisms reshape the tumor microenvironment and promote effector T cell expansion, thereby synergistically enhancing the efficacy of chemotherapy and immune checkpoint inhibitors (42, 44). On the other hand, certain pathogenic strains (such as *E. faecalis*) primarily secrete colonization factors, such as adhesins and aggregation substances, to breach host barriers. Furthermore, they directly compromise tissue integrity by secreting virulence factors such as cytolysin and gelatinase. This induces oxidative stress and DNA damage, and mediates chronic inflammation and immune evasion, ultimately acting as an “accomplice” in tumor progression (45). Therefore, in-depth elucidation of the regulatory mechanisms underlying this dynamic “commensalism–pathogenesis” balance holds important clinical translational value for developing *Enterococcus*-based combination therapeutic strategies and screening predictive biomarkers (**Table 1**).

**Table 1:** Summary of the roles, mechanisms, and clinical evidence of *Enterococcus* species in cancer.

Species of Enterococcus	Type of cancer	Effectors	Mechanism		Clinical stage	Experimental model	Level of evidence	Relevant evidence	References
			Tumor-promoting	Tumor-suppressing					
Enterococcus faecalis	Mouse colorectal cancer model	FABP2/MMPs/Plasminogen Collagen/ DDR1	DNA damage / FABP2 induction Activate MMP /plasminogen system Collagen-induced DDR1 activation		Preclinical research	Mouse model(s)	Level 3	In vitro and animal carcinogenicity Validation of associated adverse effects in clinical samples	(Jacobson et al. (59))
Enterococcus hirae	Lymphoma/Solid Tumor	bacterial translocation/ATG4B		Gut-to-lymphoid translocation CD8+ T cell activation & IFN-γ secretion Epithelial autophagy (ATG4B-dependent) Microbiota remodeling (Bifidobacterium enrichment)	Preclinical and Translational Research	Mouse tumor model(s)	Level 4	Early cohorts link it to cyclophosphamide efficacy; currently investigated as a biomarker.	(Choi et al. (46); Daillère et al. (42); Goubet et al. (48))
Enterococcus faecium	Solid Tumor Liver cancer	Specific enzyme (SagA) Bacteriocin (Enterocin)		SagA: PG Remodeling & Fragment Release Pro-inflammatory NOD2 activation Electrostatic targeting (cancer cell) Membrane permeability & depolarization Mitochondrial apoptosis (cytochrome c release)	Preclinical and Observational Studies	Mouse tumor model(s)	Level 4	Animal and in vitro data confirm sorafenib enhancement, correlating positively with clinical samples; no interventional trials exist.	(Espinosa et al. (43); Schaefer et al. (16))
Enterococcus lactis	Liver cancer/melanoma	BMVs STING pathway		Targeted BMV delivery (tumor/immune organs) STING activation (cGAS/TBK1/IRF3) Type I IFN induction (IFN-β secretion) T cell activation (antitumor activity) Metabolic synergy (overcoming PD-1 resistance)	Preclinical research	Mice / In vitro cells	Level 4	Animal studies demonstrated activation of the STING pathway; no human trial data are currently available.	(Xian et al. (53))
Enterococcus gallinarum	solid tumor	Flagellin		TLR5-NF-κB activation (flagellin-triggered innate immune signaling cascade) L-8 recruitment (epithelial inflammation & infiltratin) DC maturation (IL-12/CXCL10 release) Microenvironment remodeling (NF-κB/MAPK upregulation) Intra-host evolutionary risk (autoimmune potential)	Phase I/II	Mouse models / Phase I/II clinical trials	Level 2b	Safety was confirmed; ICI-refractory solid tumors: SD and increased immune infiltration.	(Lauté-Caly et al. (22))

The table summarizes the dual effects (tumor-promoting vs. tumor-suppressing) of specific *Enterococcus* species across different cancer types. FABP2, fatty acid-binding protein 2; MMPs, matrix metalloproteinases; DDR1, discoidin domain receptor 1; ATG4B, autophagy-related protein 4B; IFN-γ, interferon-γ; SagA, surface antigen A; PG, peptidoglycan; BMVs, bacterial membrane vesicles; STING, stimulator of interferon genes; TLR5, toll-like receptor 5; NF-κB, nuclear factor kappa-light-chain-enhancer of activated B cells; DC, dendritic cell; ICI, immune checkpoint inhibitor; SD, stable disease. The “Level of evidence” is categorized based on the study design (e.g., Level 2b for individual cohort studies, Level 3 for animal/case-control studies, Level 4 for case series or poor-quality cohort studies).

### 4.1 Mechanisms of *Enterococcus* in enhancing chemotherapeutic drug activity

Specific *Enterococcus* strains (such as *E. hirae*) activate systemic antitumor immune responses through bacterial translocation (42). These strains can actively penetrate the compromised intestinal barrier and migrate to secondary lymphoid organs, such as the spleen and lymph nodes, inducing local immune microenvironment remodeling. Furthermore, they promote pathogenic Th17 (pTh17) cell differentiation and drive the directional infiltration of CD4^+^ and CD8^+^ effector T cells into tumor sites (46). These activated tumor-specific T cells significantly enhance the body’s immune surveillance and killing capacity against tumors, synergizing chemotherapy and immunotherapy. In germ-free mice or mice treated with antibiotics to deplete Gram-positive bacteria, decreased pTh17 cells were observed, leading to resistance to cyclophosphamide (CTX). However, re-establishing the colonization of *Enterococcus faecium* can partially restore the anti-tumor efficacy of CTX (40, 42, 47). Furthermore, cyclophosphamide-induced intestinal barrier damage provides anatomical basis for *Enterococcus* translocation. The translocated strains subsequently enhance the antitumor effects of chemotherapeutic drugs by activating the Th1/Th17 immune responses (40). Researchers evaluated the *Enterococcus*-specific memory T cell responses in 38 patients with advanced lung and ovarian cancers, revealing that the magnitude of these responses was positively correlated with prolonged progression-free survival (PFS) following chemotherapy (42). These findings suggest that *Enterococcus* -induced specific immune responses can synergize with chemotherapeutic drugs to prolong patients’ PFS. This highlights the clinical potential of modulating the gut microbiome to improve cancer treatment outcomes (48).

### 4.2 *Enterococcus* sensitizes targeted therapy (using sorafenib as an example)

Clinical metagenomic analysis of the gut microbiota in patients with advanced hepatocellular carcinoma receiving sorafenib revealed a significant enrichment of *E. faecium* in patients who responded to sorafenib monotherapy (49). In mouse experiments, the anti-tumor efficacy of sorafenib was significantly enhanced by fecal microbiota transplantation (FMT) from responders or by the supplementation of E. faecium alone. Subsequent flow cytometry and other assays of the immune microenvironment in experimental mice revealed that E. faecium promotes IL-12 secretion by dendritic cells, which expands the IFN-γ+CD8+ T cell population and ultimately induces ferroptosis in tumor cells via the IFN-γ/SLC7A11/GPX4 signaling axis (49). *In vivo* mouse model experiments have also confirmed that *E. faecium* can effectively enhance the inhibitory effect of sorafenib on hepatocellular carcinoma growth (49). In conclusion, the enrichment of *E. faecium* may enhance the antitumor effects of sorafenib through the aforementioned signaling cascade. This provides a novel strategic approach for the precise clinical modulation of the gut microbiome to overcome targeted drug resistance.

### 4.3 Mechanisms by which *Enterococcus* enhances the efficacy of immune checkpoint inhibitors

The gut microbiota of patients who respond well to PD-1/PD-L1 inhibitors is frequently enriched with specific *Enterococcus* strains (such as *E. hirae* and *E.* lactis). The genome of *E. hirae* often contains integrated phage genetic elements. The phage-encoded tail tape measure protein (TMP) shares high structural similarity with tumor antigens, thereby inducing cross-reactive T cell responses. This was demonstrated in mouse experiments, wherein administration of bacterial strains expressing this TMP epitope improved the efficacy of cyclophosphamide or anti-PD-1 antibody immunotherapy (25). Gut enterococcal prophages were identified in the feces of patients with renal and lung cancers. Furthermore, the corresponding TMP cross-reactive antigens were expressed within the tumors, and their expression levels were positively correlated with the long-term clinical benefits of PD-1 blockade therapy (25). Similar findings have also been observed in patients with melanoma.

*Enterococcus* expressing the peptidoglycan remodeling enzyme secreted antigen A (SagA) releases immunologically active muramyl dipeptide (MDP) fragments through controlled cell wall turnover. These fragments significantly enhance the efficacy of immune checkpoint inhibitors (ICIs) via the NOD2 signaling pathway (16, 43). In melanoma-bearing mice, oral administration of SagA^+^ strains, resulted in a 50–80% reduction in tumor volume, significantly prolonged survival and marked expansion of intratumoral CD8^+^ cytotoxic T cells.

Furthermore, the SagA gene can be horizontally transferred across bacterial species while retaining its function. Heterologous expression and secretion of sagA in other nonpathogenic bacteria successfully preserves its anti-tumor properties (44, 50). This suggests immense clinical translational value, as it allows for the introduction of the target gene into well-characterized safe probiotic chassis (such as *Lactobacillus* and *Escherichia coli Nissle 1917*), thereby circumventing the potential pathogenicity and antibiotic resistance risks associated with *Enterococcus*. Administration of synthetic MDP-l, d analogs alone is sufficient to induce T cell infiltration and the proliferation of M1-type monocytes. This suggests that future strategies could utilize synthetic molecules to mimic microbial signals, thereby circumventing the pathogenic risks associated with live bacteria and safely activating antitumor immunity (51).

*Enterococcus lactis* MNC-168 can serve as a live biotherapeutic product (LBP) in a murine MC-38 colorectal cancer subcutaneous xenograft model. MNC-168 releases bacterial membrane vesicles (BMVs) that specifically target and accumulate in tumor tissues and the spleen. These BMVs trigger the cGAS/STING/TBK1/IRF3 signaling pathway, thereby promoting the production of interferon-β (IFN-β), a key molecule in innate immunity (52). Consequently, this pathway enhances the infiltration of IFN-γ+ CD4+ and IFN-γ+ CD8+ T cells within the tumor microenvironment. Multiple preclinical cancer models, such as the *MC-38* colorectal cancer model, have demonstrated that *MNC-168* inhibits tumor growth and enhances the efficacy of anti-PD-1 immune checkpoint blockade (ICB) therapy. Building on these preclinical findings, this strain has been developed into an innovative enteric-coated capsule formulation. A Phase I clinical trial for treating advanced malignant solid tumors has been approved by the Australian Human Research Ethics Committee (53).

### 4.4 *Enterococcus*-derived immunomodulators

Specific *Enterococcus* strains (*e.g.*, *E. faecium*) secrete bacteriocins with direct antitumor activity. For instance, *Enterocin* 12a selectively inhibited the proliferation of various human cancer cell lines in a dose-dependent manner, but exhibited no such effect on normal human peripheral blood mononuclear cells, as determined by the MTT assay. Cancer cells treated with *Enterocin* 12a exhibit apoptosis-like morphological changes (54), demonstrating that *Enterococcus*-secreted bacteriocins possess the potential to induce apoptosis in tumor cells.

*Enterococcus gallinarum* MRx0518 is a candidate live biotherapeutic with proven antitumor efficacy. Studies have suggested that prophylactic oral administration of this strain produces robust antitumor effects in multiple mouse cancer models, such as breast, lung, and renal cancer. Its flagellin enhances the cytotoxic activity of tumor-specific CD8+ T cells and promotes the production of antigen-specific IFN-γ via TLR5 stimulation, thereby augmenting the antitumor immune response. Studies have found that *E. gallinarum* is enriched in the feces of patients who respond to anti-PD-1 therapy. This suggests that this species and its expressed flagellin may facilitate the responsiveness of patients to immune checkpoint inhibitor (ICI) treatment (22). A Phase I/II clinical trial for advanced solid tumors is currently underway (NCT03637803). Interim results imply that the combination of MRx0518 and anti-PD-1 therapy exhibits a favorable safety and tolerability profile without inducing dose-limiting toxicities, demonstrating a favorable safety profile for oral administration in humans. Signs of tumor shrinkage or stable disease (SD) were observed in some patients with primary resistance to PD-1 inhibitors. This suggests that MRx0518 has the potential to serve as an “adjuvant” to help restore the body’s sensitivity to immunotherapy.

### 4.5 Direct promotion of cancer initiation and progression by *Enterococcus*

Unlike the aforementioned bacterial species, *E. faecalis* has demonstrated protumorigenic characteristics in previous studies. The abundance of *E. faecalis* in fecal samples from colorectal cancer patients is higher than that in healthy controls. It may directly drive tumorigenesis by inducing genotoxic damage and remodeling the tumor microenvironment (55). Animal experiments have shown that intragastric administration of *E. faecalis* significantly accelerates orthotopic tumor growth and increases the number of liver metastases. *E. faecalis* metabolically generates large amounts of reactive oxygen species (ROS) that directly induce the DNA in colonic epithelial cells. The continuous accumulation of DNA damage drives the malignant transformation of normal cells (45). The *E. faecalis* metabolite biliverdin activates the PI3K/AKT/mTOR signaling pathway, promoting colorectal cancer cell proliferation and tumor angiogenesis (56). Furthermore, it accelerates tumorigenesis and progression by activating oncogenic signaling molecules such as CDK8 (57, 58).

### 4.6 Multiple mechanisms of *E. faecalis* in promoting tumor invasion and distant metastasis

In colorectal cancer models, comparison between wild-type *Enterococcal strains (V583, GelE^+^) a*nd their mutants revealed no significant difference in overall tumor burden. However, the wild-type group exhibited substantial remodeling of the intratumoral immune microenvironment, presenting an immunosuppressive state favorable for tumor growth. This phenomenon may be attributed to collagen degradation products activating the DDR1 signaling pathway in colorectal cancer cells. The cascade involving extracellular matrix degradation, DDR1 activation, and subsequent immune microenvironment remodeling constitutes the core mechanism by which *Enterococcal strain (V583, GelE^+^)* fosters a microenvironment conducive to tumor dissemination (59).

Multi-omics profiling of 242 tumor nodules in 58 patients with multifocal hepatocellular carcinoma (HCC) indicated significant enrichment of *Enterococcus* (e.g., *E. faecalis*) in intrahepatic metastatic nodules. Both *in vitro* and *in vivo* experiments have confirmed that bacterial species such as *E. faecalis* promote the migration, invasion, and pulmonary metastasis of HCC cells. This is achieved by inducing an immunosuppressive microenvironment (e.g., increased MDSCs and impaired CD8+ T cell function) and activating the epithelial-mesenchymal transition (EMT) (60–62).

## 5 Clinical safety translation of Enterococcus

Although Enterococcus holds promise as delivery vehicles or synergistic adjuvants for immune checkpoint inhibitors (ICIs), the primary bottleneck in the clinical translation of *Enterococcus* is precisely balancing immune activation efficacy against opportunistic pathogenesis risks, necessitating strict biosafety evaluations and regulatory frameworks. Studies have suggested that several specific strains (*e.g.*, MRx0518 and SF68) exhibit efficacy in modulating the immune microenvironment, reversing PD-1 inhibitor resistance, and improving metabolic profiles (22). Furthermore, in early-phase Phase I/II clinical trials (NCT03637803), the combination of this strain with anti-PD-1 therapy indicated a favorable safety profile and tolerability. Beyond live bacterial preparations, phage therapy targeting *Enterococcus* is also under development. Studies have suggested that specific bacteriophages exhibit significant antibacterial and antibiofilm activities against multidrug-resistant enterococci, including vancomycin-resistant *Enterococcus* (VRE), in animal models, holding promise as an alternative therapeutic strategy in the post-antibiotic era (63). Nevertheless, the inherent opportunistic pathogenicity of *Enterococcus* (particularly its propensity to cause bloodstream infections) and the increasingly severe issue of antibiotic resistance (*e.g.*, VRE and HLAR) constitute major safety concerns (**Table 2**).

**Table 2:** Assessment of the transformation potential and safety challenges of *Enterococcus* species in oncology.

Species of Enterococcus	Conversion potential rating	Core application scenarios	Key security challenges
E. faecalis	☆☆	Early screening targets and blocker development for colorectal cancer	Cancer promotion, DNA damage, sepsis
E. hirae	☆☆☆☆	Combination Chemotherapy and Effectiveness Prediction	Bacterial translocation leads to bacteremia and VRE resistance
E. faecium	☆☆☆	Antimicrobial peptide drugs, NOD2 agonists	Strain-specific virulence and antibiotic resistance gene transfer
E. lactis	☆☆☆☆	STING pathway activation and PD-1 combination therapy	Relatively safe, need to keep an eye on inflammation levels
E. gallinarum	☆☆☆☆☆	Refractory solid tumors, oncolytic immunotherapy	Autoimmune reactions, acute infusion reactions

The table summarizes the core application scenarios and associated risks for five *Enterococcus* species. PD-1, programmed cell death protein 1; STING, stimulator of interferon genes; NOD2, nucleotide-binding oligomerization domain-containing protein 2; VRE, vancomycin-resistant *Enterococcus*. The “Conversion Potential Rating” is based on a 5-star scale, where ☆ represents the lowest potential and ☆☆☆☆☆ represents the highest potential for clinical translation and therapeutic application.

### 5.1 Strict genomic screening and virulence evaluation

First, precise identification at the strain level is essential. Prior to clinical translation, whole-genome sequencing must be employed to screen for and exclude strains carrying transferable antibiotic resistance genes (e.g., VanA/VanB-type vancomycin resistance genes) or functional virulence factors (e.g., hemolysin and gelatinase). Blocking horizontal gene transfer at the genetic source prevents live biotherapeutic products (LBPs) from serving as reservoirs or vectors for resistance genes (64, 65). Second, engineering modifications through synthetic biology approaches include (66, 67), for example, transferring the SagA gene from *Enterococcus* to safer probiotic strains, deletion of cell wall modification genes (*e.g.*, epa and dlt) to decrease gut colonization, or incorporating an anaerobe-specific promoter to guarantee automatic gene silencing upon translocation into aerobic environments (*e.g.*, the bloodstream) (68). Additionally, an intrinsic immune feedback mechanism (e.g., PD-L1 expression) can be engineered to prevent immune-related adverse events, while pre-set emergency clearance protocols based on suicide switches (e.g., the *araC-PBAD* system) provide a fail-safe mechanism (69, 70). Finally, for high-risk patients, live bacteria-free alternatives are preferred to bypass the risks associated with live bacteria (71). For instance, *Enterococcus* outer membrane vesicles (OMVs) or inactivated bacteria as ICI delivery vehicles (72). Gao et al. (73) proposed the remote loading of drugs via a pH gradient between the inner and outer membranes of bacterial membrane vesicles (BMVs), successfully increasing the drug loading capacity to 12%.

### 5.2 Precise stratification and risk control in clinical application

Strict subject stratification is required in clinical applications; high-risk groups (e.g., immunosuppressed, granulocytopenic, or catheterized patients) require close monitoring for bacteremia. Antibiotic treatment should shift from broad-spectrum coverage to precision intervention: selectively targeting protumorigenic or resistant clones while restoring antitumor probiotics (e.g., via fecal microbiota transplantation [FMT] or specific formulations) to prevent microbiome disruption caused by indiscriminate antibiotic use (74).

### 5.3 Full-cycle dynamic monitoring and adaptive regulation

Establishment of a full-lifecycle regulatory framework spanning the laboratory, clinical, and post-marketing stages is essential. During clinical trials, alongside routine dose-limiting toxicity (DLT) monitoring, dynamic blood cultures are recommended to track bacteremia. Efficacy assessment requires integrating rapid diagnostics to differentiate between colonization and active infection (75). Although relatively effective biomedical applications have been developed, rigorous methods are still needed to generate reproducible and scalable translational drug delivery systems. Furthermore, establishing a robust pharmacovigilance system to dynamically monitor strain colonization stability and resistance phenotype drift is necessary, ensuring that infection and resistance risks remain acceptable while fully leveraging its immune adjuvant value.

## 6 Conclusion

This review summarizes recent advances in enterococcal-mediated antitumor immunity. However, most breakthrough findings—such as the role of SagA—are currently limited to preclinical models, which cannot fully replicate the complexity of human diets, genetics, and microbiomes. In addition, most existing human data are based on retrospective observational studies, which are highly susceptible to confounding factors. Therefore, the claim regarding “improved efficacy” should currently be regarded as a scientifically plausible hypothesis with biological rationale, rather than an established clinical fact. Moving forward, these findings must be validated through rigorous randomized controlled trials (RCTs) to establish their level of evidence.

*Enterococci* exert a profoundly context-dependent and strain-specific duality in cancer therapy, functioning either as potentiators of immunotherapeutic efficacy or as catalysts for tumorigenesis. Different *Enterococcus* strains differentially modulate the immune system, exhibiting corresponding proinflammatory or anti-inflammatory capacities. Specific strains (*e.g.*, *E. faecalis, E. hirae, E. lactis*) activate systemic antitumor immunity via SagA-mediated NOD2 signaling, vesicle-driven STING activation, or secreted extracellular polysaccharides, thereby enhancing cyclophosphamide activity, sorafenib sensitivity, and the efficacy of immune checkpoint inhibitor (ICIs). Conversely, pathogenic E. *faecalis* drives tumorigenesis, invasion, and metastasis in specific microenvironments (*e.g.*, colorectal cancer) by inducing oxidative stress and DNA damage, activating MMP-9 and the plasminogen system, and fostering an immunosuppressive microenvironment (57, 59). The anti-tumor effects of *Enterococcus* have been validated in multiple preclinical studies. *Enterococcus*-based formulations are expected to synergize with anti-tumor agents by modulating the gut microbiome, thereby improving patient prognosis and overall survival (53). Nevertheless, the pathogenicity and antimicrobial resistance of *Enterococcus*, as an opportunistic pathogen, pose urgent challenges for its clinical translation. The feasibility of clinical translation is enhanced by heterologous sagA expression and enteric-coated vesicle capsules (76). Currently, *E. lactis* MNC-168 and *E. gallinarum* MRx0518 are in clinical trials, showing preliminary synergistic anti-tumor efficacy (22). Future studies should elucidate strain-specific functional heterogeneity and identify biomarkers distinguishing beneficial from harmful strains. Integrating individual microbiome profiles to enable precise regulation—via probiotic colonization, genetic engineering, or selective depletion—will maximize therapeutic benefits while minimizing pro-tumorigenic risks, ushering in an era of “microbiome-empowered” cancer therapy.
